# HTRA1 Mutations Identified in Symptomatic Carriers Have the Property of Interfering the Trimer-Dependent Activation Cascade

**DOI:** 10.3389/fneur.2019.00693

**Published:** 2019-06-28

**Authors:** Masahiro Uemura, Hiroaki Nozaki, Akihide Koyama, Naoko Sakai, Shoichiro Ando, Masato Kanazawa, Taisuke Kato, Osamu Onodera

**Affiliations:** ^1^Department of Neurology, Brain Research Institute, Niigata University, Niigata, Japan; ^2^Department of Medical Technology, Graduate School of Health Sciences, Niigata University, Niigata, Japan; ^3^Division of Legal Medicine, Niigata University, Niigata, Japan; ^4^Department of System Pathology for Neurological Disorders, Brain Research Institute, Niigata University, Niigata, Japan

**Keywords:** heritability, vascular dementia, mutations, HTRA1, carriers, CARASIL

## Abstract

**Background:** Mutations in the *high-temperature requirement A serine peptidase 1 (HTRA1)* cause cerebral autosomal recessive arteriopathy with subcortical infarcts and leukoencephalopathy (CARASIL). Most carriers for *HTRA1* mutations are asymptomatic, but more than 10 mutations have been reported in symptomatic carriers. The molecular differences between the mutations identified in symptomatic carriers and mutations identified only in CARASIL patients are unclear. HTRA1 is a serine protease that forms homotrimers, with each HTRA1 subunit activating the adjacent HTRA1 via the sensor domain of loop 3 (L3) and the activation domain of loop D (LD). Previously, we analyzed four HTRA1 mutant proteins identified in symptomatic carriers and found that they were unable to form trimers or had mutations in the LD or L3 domain. The mutant HTRA1s with these properties are presumed to inhibit trimer-dependent activation cascade. Indeed, these mutant HTRA1s inhibited wild-type (WT) protease activity. In this study, we further analyzed 15 missense HTRA1s to clarify the molecular character of mutant HTRA1s identified in symptomatic carriers.

**Methods:** We analyzed 12 missense HTRA1s identified in symptomatic carriers (hetero-HTRA1) and three missense HTRA1s found only in CARASIL (CARASIL-HTRA1). The protease activity of the purified recombinant mutant HTRA1s was measured using fluorescein isothiocyanate-labeled casein as substrate. Oligomeric structure was evaluated by size-exclusion chromatography. The protease activities of mixtures of WT with each mutant HTRA1 were also measured.

**Results:** Five hetero-HTRA1s had normal protease activity and were excluded from further analysis. Four of the seven hetero-HTRA1s and one of the three CARASIL-HTRA1s were unable to form trimers. The other three hetero-HTRA1s had mutations in the LD domain. Together with our previous work, 10 of 11 hetero-HTRA1s and two of six CARASIL-HTRA1s were either defective in trimerization or had mutations in the LD or L3 domain (*P* = 0.006). By contrast, eight of 11 hetero-HTRA1s and two of six CARASIL-HTRA1 inhibited WT protease activity (*P* = 0.162).

**Conclusions:** HTRA1 mutations identified in symptomatic carriers have the property of interfering the trimer-dependent activation cascade of HTRA1.

## Introduction

Loss-of-function mutations in the *high-temperature requirement A serine peptidase 1 (HTRA1)* gene cause cerebral autosomal recessive arteriopathy with subcortical infarcts and leukoencephalopathy (CARASIL) (1, 2). HTRA1 is a serine protease that forms a homotrimer. The sensor domain of loop 3 (L3) and the activation domain of loop D (LD) play essential roles in trimer-mediated activation of the neighboring HTRA1 (3, 4).

Recently, symptomatic carriers have been reported for several *HTRA1* mutant alleles (5–9). We analyzed four *HTRA1* alleles identified in symptomatic carriers and found that the proteins inhibited WT protease activity (6). In contrast, two of three missense HTRA1s observed only in CARASIL patients did not inhibit WT protease activity. Therefore, we proposed that the missense HTRA1s identified in symptomatic carriers have unique molecular characteristics (6). However, these characteristics have not been evaluated in other missense HTRA1s identified in symptomatic carriers or in homozygous CARASIL patients. The prediction of the pathogenicity of HTRA1 mutation in carriers is important to relatives of homozygous CARASIL patients, and carriers for variant *HTRA1* alleles. In this study, we have evaluated an additional 15 missense HTRA1 mutants identified in symptomatic carriers and CARASIL patients.

## Methods

### Mutation Selection

From a literature search performed up to December 2017, we retrieved 12 missense *HTRA1* alleles identified in symptomatic carriers: S121R, A123S, R133G, R166C, R166L, A173P, S284G, S284R, P285Q, F286V, G295R, and D450H; and 3 missense *HTRA1* alleles reported only in CARASIL patients: A173T, A321T, and L364P (5, 8–12). Clinical characteristics of the symptomatic carriers are summarized in Supplemental Table 1. This study was approved by the Niigata University institutional review board.

### Measurement of HTRA1 Protease Activity

**Figure 1:**
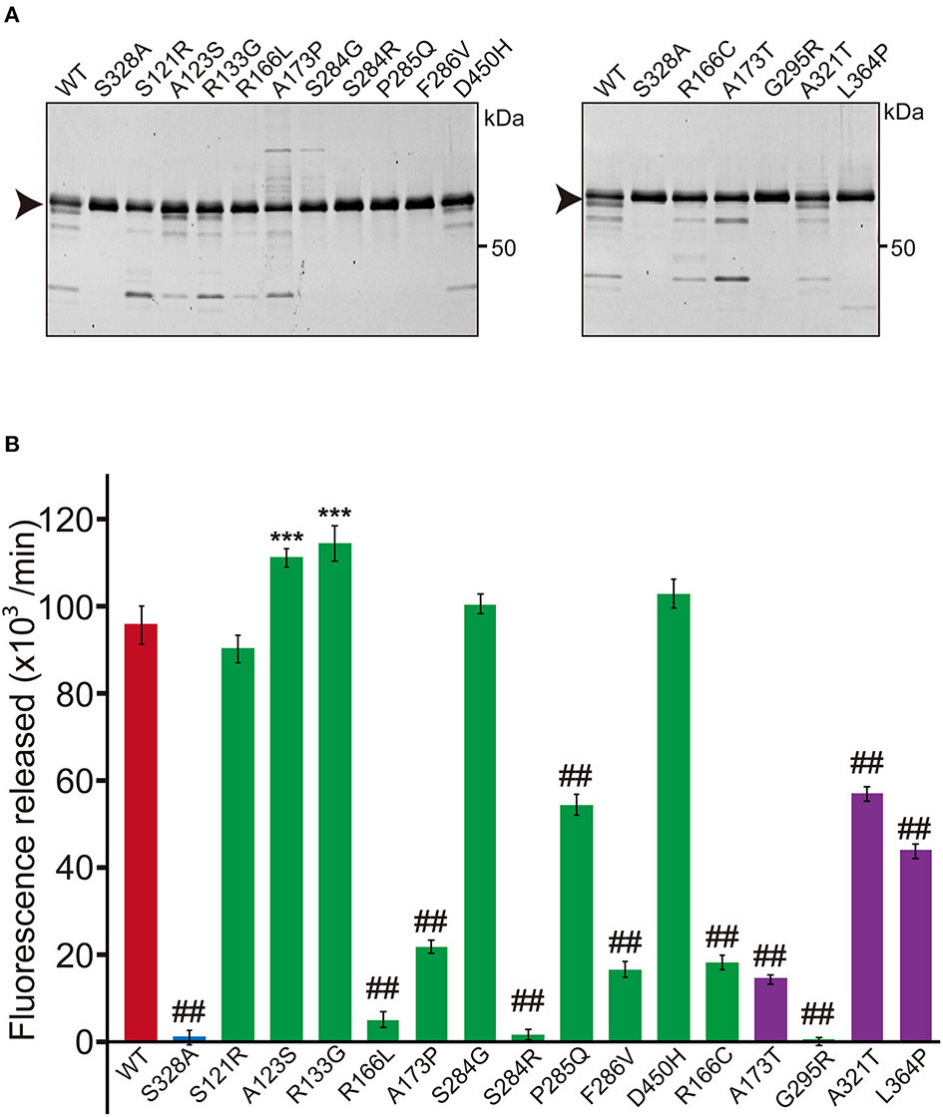
Protease activity of missense HTRA1s identified in symptomatic carriers. **(A)** SDS-PAGE of WT and missense mutant HTRA1 proteins used in the protease assay. Black arrows indicate the full-length band of HTRA1 tagged with myc-His_6_. **(B)** Protease activities of missense HTRA1s identified in symptomatic carriers and CARASIL patients. Activities were calculated from the slope of the linear portion of the normalized fluorescence vs. time (30, 60, 90 min) plots. Mean values from 3 independent experiments are shown. Red and blue bars indicate protease activities of WT and S328A, the positive and negative controls, respectively. Green bars indicate protease activities of missense HTRA1s identified in symptomatic carriers. Purple bars indicate protease activities of missense HTRA1s identified only in CARASIL patients. I-bars indicate standard errors (SE). Statistical comparisons of protease activities between WT and each missense HTRA1 protein were performed with one-way analysis of variance followed by the Dunnett's *post hoc* test. ^***^P < 0.0001 for protease activities of each HTRA1 relative to WT. ^##^*P* < 0.0001 for protease activities of HTRA1 relative to WT.

An expression plasmid for each variant *HTRA1* cDNA was generated using the GENEART® Site-Directed Mutagenesis System (Invitrogen, Carlsbad, CA). The WT-*HTRA1* cDNA tagged with a C-terminal myc-His_6_ was subcloned into the pcDNA 3.1 vector (Invitrogen) was the substrate for mutagenesis. The concentration of each plasmid was measured using the Quant-iT^TM^ PicoGreen dsDNA Assay Kit (Thermo Fisher Scientific, Waltham, MA, USA). The *HTRA1* cDNA plasmid vectors were transfected into FreeStyle 293 cells (Thermo Fisher Scientific) and incubated for 72 h. After incubation, secreted HTRA1 protein was purified from the culture medium using a HisTrap FF crude column (GE Healthcare, Cleveland, OH, USA). The concentration of recombinant HTRA1 proteins was measured using a Bicinchoninic Acid (BCA) Protein Assay Kit (Wako, Osaka, Japan). After pre-incubating 1 μg of the recombinant HTRA1 protein, protease activities were evaluated at 37°C using fluorescein isothiocyanate (FITC)–labeled casein as a substrate (Fluorescent Protease Assay Kit; Pierce, Rockford, IL, USA) (2, 6). WT was used as the positive control and S328A, which is deficient in protease activity, was used as the negative control. Fluorescence was measured using a FilterMax F5 Multi-Mode Microplate Reader (Molecular Devices, Sunnyvale, CA, USA). Protease activities were calculated from the slope of the linear portion of the normalized fluorescence vs. time plots at 30, 60, and 90 min using MATLAB® R2017b (9.3.0.713579). The amount of each HTRA1 protein was analyzed by sodium dodecyl sulfate polyacrylamide gel electrophoresis (SDS-PAGE), stained by SYPRO® Ruby Protein Gel Stain (Thermo Fisher Scientific). Five mutations, S121R, A123S, R133G, S284G, and D450H, were excluded from further analysis because the protease activities of these mutations were normal (Figure 1B); two of these five mutations were appeared to be benign (A123S and R133G) (5).

### Analysis of Oligomerization of HTRA1 Proteins

**Figure 2:**
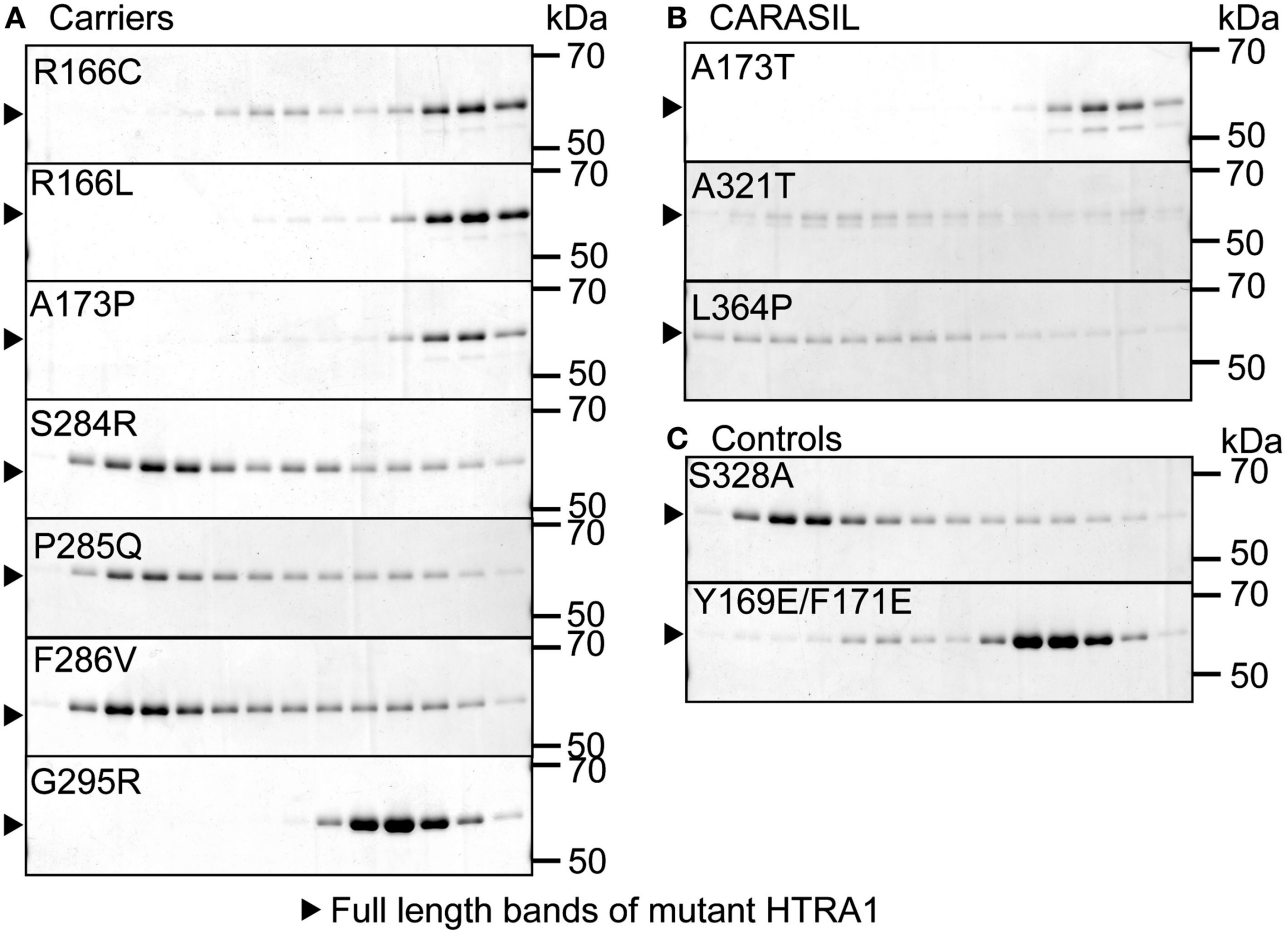
Oligomerization of missense HTRA1s. Size-exclusion chromatography of mutant and WT HTRA1 protein preparations. Fractions were separated by SDS-PAGE and visualized by Coomassie Brilliant Blue staining. **(A)** Oligomeric state analysis of each missense HTRA1 identified in symptomatic carriers. **(B)** Oligomeric state analysis of each missense HTRA1 found only in CARASIL. **(C)** Oligomeric state analysis of S328A and Y169E/F171E HTRA1.

Oligomerization was evaluated by size-exclusion chromatography using a Superdex 200 10/300 GL column (GE Healthcare, Chicago, IL, USA) on an AKTA FPLC workstation equilibrated with Tris-buffered saline (100 mM Tris-HCl, pH 8.0 and 150 mM NaCl). After pre-incubation at 37°C for 30 min, each HTRA1 protein sample was injected at 500 ng/μl. Y169E/F171E HTRA1, an artificial, known monomeric HTRA1, was used as the reference monomer and S328A HTRA1 was used as a reference trimer (Figure 2C) (4, 6). SDS-PAGE was used to evaluate fractions from size-exclusion chromatography (6).

### Evaluation of Dominant-Negative Effects of HTRA1 Mutants on Wild-Type HTRA1

Each *HTRA1* variant vector was cotransfected with an equal amount of WT vector into FreeStyle 293 cells. Purification of the mixed HTRA1 proteins and evaluation of their protease activity were performed as described above. We compared the protease activities of the mixtures of WT with each variant HTRA1 to that of a mixture of WT and S328A, an artificially inactive HTRA1 that forms a trimer with WT and does not have a dominant-negative effect; this was used as a reference to adjust the protein concentration in the reaction mixture (6). The mixtures of G283E with WT and A252T with WT were used as positive and negative controls for dominant-negative effects, respectively (6).

### Statistical Analysis

Statistical analyses were performed using R 3.2.2. Groups were compared using one-way analysis of variance (ANOVA) for independent samples, followed by Dunnett's multiple-comparison test when overall *P* was < 0.05. Fisher's exact test was used to compare the frequencies of mutant HTRA1s with dominant-negative effect between symptomatic carriers and CARASIL.

## Results

### Oligomerization of Missense HTRA1 Mutant Proteins

Five missense HTRA1s (S121R, A123S, R133G, S284G, and D450H) showed normal protease activity, thus we excluded these HTRA1s from further analysis (Figures 1A,B). Protease activities of other HTRA1s were significantly decreased relative to WT activity. We assayed trimerization of the other HTRA1s and found that four hetero-HTRA1s (R166C, R166L, A173P, and G295R) and one CARASIL-HTRA1 (A173T) were unable to form trimers (Figures 2A,B). Among mutant HTRA1s form trimers, all hetero-HTRA1s had mutations in the LD domain (residues 284–290) or L3 loop domain, (residues 301–314) while two CARASIL-HTRA1s had mutations in the protease domain (residues 204–356) (Table 1).

**Table 1:** Summary information of previously-reported *HTRA1* mutations identified in symptomatic carriers and CARASIL patients.

	**Amino acid substitution**	**Location (Domain)**	**Trimerization**	**Dominant-negative**	**References**
Reported in symptomatic carriers hetero-HTRA1s	**R166L**	Other	Defective	+	(5)
	**R166C**	Other	Defective	–	(8)
	**A173P**	Other	Defective	+	(5)
	G283E*	Protease	Defective	+	(6)
	**G295R**	Protease	Defective	+	(9)
	T319I*	Protease	Defective	+	(6)
	**S284R**	LD	Trimer	+	(5)
	P285L*	LD	Trimer	+	(6)
	**P285Q**	LD	Trimer	–	(5)
	**F286V**	LD	Trimer	–	(5)
	R302Q*	L3	Trimer	+	(6)
	*R302X*	L3	u.d.	u.d.	(7)
Reported only in CARASIL CARASIL-HTRA1s	**A173T**	Other	Defective	+	(12)
	R274Q*	Protease	Defective	+	(6)
	A252T*	Protease	Trimer	–	(6)
	V297M*	Protease	Trimer	–	(6)
	**A321T**	Protease	Trimer	–	(11)
	**L364P**	Protease	Trimer	–	(10)
	*E42Dfs*	IGFBP	u.d.	u.d.	(11)
	*G56Afs*	IGFBP	u.d.	u.d.	(13)
	*K168X*	Other	u.d.	u.d.	(14)
	*E247Rfs*	Protease	u.d.	u.d.	(14)
	*E277Vfs*	Protease	u.d.	u.d.	(14)
	*R370X*	Protease	u.d.	u.d.	(2)

*Asterisks: mutant HTRA1s evaluated in our previous report (6). Bold font: mutant HTRA1s analyzed in this study. Italics: frameshift or non-sense mutations. IGFBP, insulin-like growth factor binding protein domain. Protease, protease domain outside the LD or L3 domain. LD, loop D domain; L3, loop 3 domain. u.d., undetermined. The mRNA of these mutants was degraded. +, present and –, absent*.

### Inhibition of WT Protease Activity

**Figure 3:**
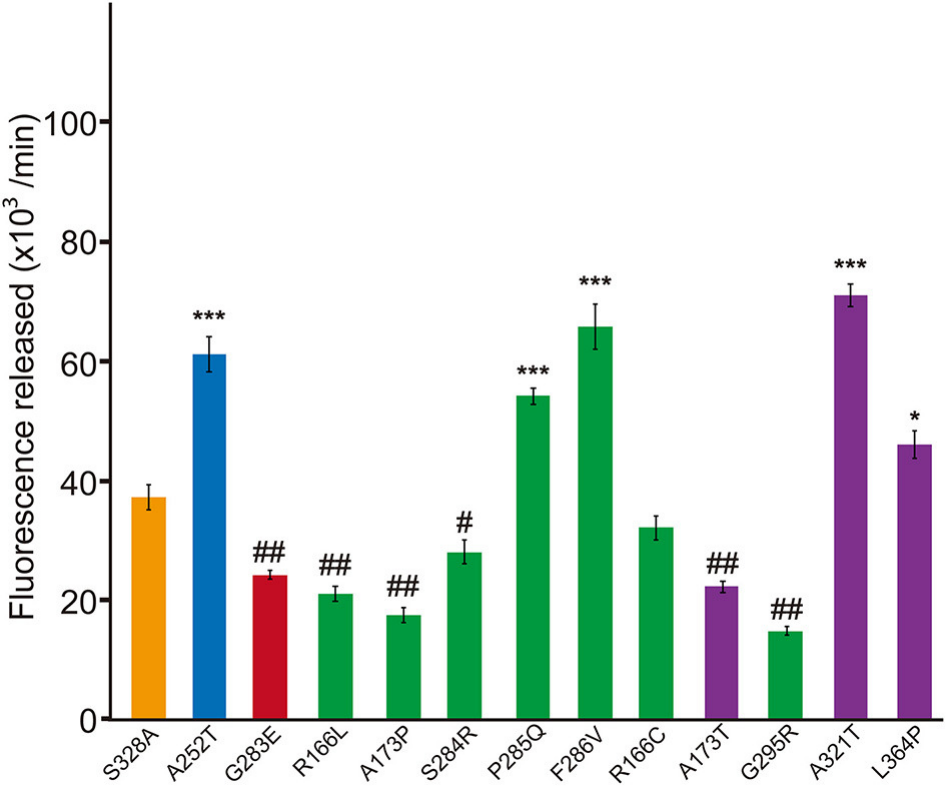
Dominant-negative effects of missense HTRA1s identified in symptomatic carriers. Protease activities of mixtures of each missense HTRA1 with WT calculated from the slope of the linear portion of normalized fluorescence vs. time (30, 60, and 90 min) plots. Orange, S328A/WT, a positive control for a dominant-negative effect. Blue and red bars indicate protease activities of A252T/WT and G283E/WT, respectively, negative and positive controls for dominant-negative effect, respectively. Green bars, missense HTRA1s identified in symptomatic carriers. Purple bars, missense HTRA1s found only in CARASIL patients. I-bars indicate standard errors (SE). Statistical comparisons of protease activities between each mutant HTRA1/WT and S328A/WT were performed with one-way analysis of variance followed by Dunnett's *post hoc* test. ^***^P < 0.0001; ^*^P < 0.05 for increases in protease activities for each HTRA1/WT relative to S328A/WT. ^##^*P* < 0.0001; ^#^*p* < 0.05 for differences for HTRA1/WT mixtures relative to S328A/WT.

Next we investigated whether these missense HTRA1s have dominant-negative. To serve as a control for this assay, the half dose of WT was not suitable because protein and substrate concentrations in the reaction mixture differ from those expressing WT and each mutant HTRA1 (6). Therefore, we used a mixture of WT and S328A, an artificial inactive HTRA1 that trimerizes, as a control (3, 4) and compared the protease activities of the mixtures of WT and each mutant HTRA1 with that of the control. dominant-negative was then defined as a protease activity less than that of the control. Four of six hetero-HTRA1s and one of four CARASIL-HTRA1 showed dominant-negative (Figure 3 and Supplemental Figure 1).

### Molecular Characteristics of Missense HTRA1 Mutant Proteins in Symptomatic Carriers

The results showed the possibility that the HTRA1 mutant proteins identified in the symptomatic carriers cannot form trimers or have mutations in the LD or L3 domain. Therefore, we examined the frequency of mutant HTRA1s with these characteristics between missense mutations identified in symptomatic carriers and only in CARASIL. Combined with our previous results, all missense HTRA1s identified in symptomatic carriers were defective in trimerization or had mutations in the LD or L3 domain, but only two of six missense HTRA1s found only in CARASIL patients were defective in trimerization and none had mutations in the LD or L3 domain (*P* = 0.006; Table 1). In contrast, the frequency of mutant HTRA1s with dominant-negative effects in symptomatic carriers was not significantly higher than that in CARASIL patients (72.7 vs. 33.3%, *P* = 0.162; Table 1).

## Discussion

In this study, we found that missense HTRA1 mutations identified in symptomatic carriers display two characteristics: defective trimerization or mutations in the LD or L3 domain. We newly identified that four HTRA1 missense mutants identified in symptomatic carriers were defective trimerization (R166C, R166L, A173P, and G295R). We also demonstrated that monomeric HTRA1 inhibits WT protease activity (6). Thus, these mutants have a more deleterious effect on carriers relative to mutants that did not inhibit WT protease activity, resulting in a symptomatic carrier. Although A173T and R274Q were also defective trimerization, no symptomatic carriers with A173T or and R274Q have been identified to date (12, 15), carriers with these mutations should be carefully evaluated for cerebral small vessel disease (CSVD).

Among the other missense HTRA1s observed in the symptomatic carriers, four of the five missense HTRA1s had a mutation in the LD domain. The simulation analysis revealed that these mutations induced an instability in the helical structure of the LD loop (16). For the L3 domain, R302 is the only portion observed in the symptomatic carrier. R302 is essential for inter-monomer communication and substrate binding (16). Thus, a mutation in the LD or L3 domain that interferes with signal transduction between the monomers result in inhibition of the WT activity.

Regarding the molecular pathogenesis in symptomatic carriers, we hypothesized that these mutations inhibit the WT protease activity by interfering with the trimer-dependent activation cascade, resulting in <50% of protease activity in symptomatic carriers (6). However, the data from this study show that not all mutations identified in symptomatic carriers showed <50% of protease activity. Moreover, symptomatic carriers with non-sense or frameshift mutations have also been reported (7, 17). Thus, we consider that the residual HTRA1 protease activity might correlate with the risk for developing CSVD. To elucidate the precise correlation between HTRA1 protease activity and risk of CSVD, we and others must identify and evaluate additional carriers for HTRA1 mutations.

Our study has several limitations. First, protease activity was measured by using casein, a non-physiological substrate. Several substrates, including fibronectin and transforming growth factor-beta binding protein 1 have been reported (18, 19). Thus, results of protease assays may be different if other substrates are used. Second, the pathogenicity of mutations outside the protease domain remains unknown. Several studies have investigated the role of the Kazal-like and PDZ domains in regulating the protease activity of HTRA1s (20–22). The Kazal-like domain has been implicated in autolysis (21), and mutations in this domain could influence its expression. Thus, the possibility that a mutation may decrease the amount of protein *in vivo* by decreasing stability or secretion to the extracellular matrix cannot be excluded.

## Conclusion

We found that a either a deficiency in trimerization or location of the amino-acid mutation in the LD or L3 domain was highly observed in individual missense *HTRA1* alleles in symptomatic carriers. Our findings will have significant utility for improving genetic counseling both for the relatives of CARASIL patients and for carriers with *HTRA1* variants with sporadic CSVDs.

## Data Availability

The raw data supporting the conclusions of this manuscript will be made available by the authors, without undue reservation, to any qualified researcher.

## Author Contributions

MU: draft of manuscript, study concept and design, acquisition of data and analysis. HN: revision of manuscript, interpretation of data, and study supervision. NS, SA, and MK: revision of manuscript and interpretation of data. AK and TK: acquisition of data and interpretation of data. OO: revision of the manuscript, study concept and design, and study supervision.

### Conflict of Interest Statement

OO has received speaking honoraria from Kyowa Hakko Kirin Co., Ltd., Bristol-Myers Squibb, Ono Pharmaceutical Co., Ltd., Mitsubishi Tanabe Pharm, Takeda, Daiichi-Sankyo, FUJIFILM, SANOFI, and FP-pharm. The remaining authors declare that the research was conducted in the absence of any commercial or financial relationships that could be construed as a potential conflict of interest.

## Supplementary Material

Supplemental Figure 1 Mixtures of missense HTRA1 proteins with WT protein. Mixture of each missense HTRA1 and WT protein evaluated by SDS-PAGE stained with SYPRO® Ruby. Broken arrows indicate the full-length band of missense HTRA1s tagged with myc-His_6_. Arrowheads indicate the full-length band of WT HTRA1 tagged with His_6_.

Supplemental Table 1 Summary of the clinical features of symptomatic heterozygotes with HTRA1 mutations.
