# Corrigendum: HTRA1 Mutations Identified in Symptomatic Carriers Have the Property of Interfering the Trimer-Dependent Activation Cascade

**DOI:** 10.3389/fneur.2021.756038

**Published:** 2021-09-08

**Authors:** Masahiro Uemura, Hiroaki Nozaki, Akihide Koyama, Naoko Sakai, Shoichiro Ando, Masato Kanazawa, Taisuke Kato, Osamu Onodera

**Affiliations:** ^1^Department of Neurology, Brain Research Institute, Niigata University, Niigata, Japan; ^2^Department of Medical Technology, Graduate School of Health Sciences, Niigata University, Niigata, Japan; ^3^Division of Legal Medicine, Niigata University, Niigata, Japan; ^4^Department of System Pathology for Neurological Disorders, Brain Research Institute, Niigata University, Niigata, Japan

**Keywords:** heritability, vascular dementia, mutations, HTRA1, carriers, CARASIL

In the original article, there were mistakes in Figures 1 and 3 as published. The multiplier of the unit for protease activity was incorrect. The correct value is 10 to the third power. The corrected Figures 1 and 3 appear below.

**Figure 1:**
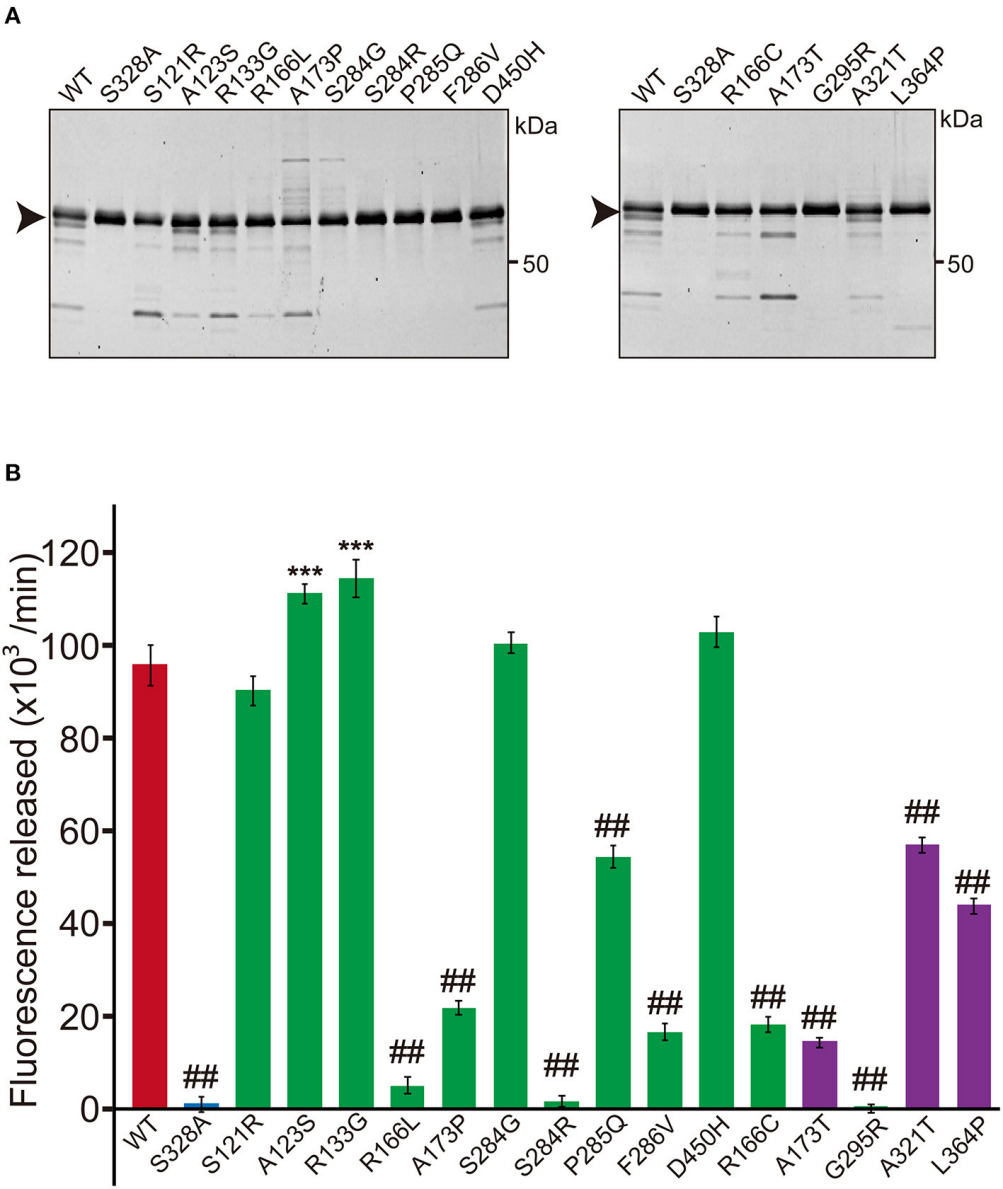
Protease activity of missense HTRA1s identified in symptomatic carriers. **(A)** SDS-PAGE of WT and missense mutant HTRA1 proteins used in the protease assay. Black arrows indicate the full-length band of HTRA1 tagged with myc-His_6_. **(B)** Protease activities of missense HTRA1s identified in symptomatic carriers and CARASIL patients. Activities were calculated from the slope of the linear portion of the normalized fluorescence vs. time (30, 60, 90 min) plots. Mean values from 3 independent experiments are shown. Red and blue bars indicate protease activities of WT and S328A, the positive and negative controls, respectively. Green bars indicate protease activities of missense HTRA1s identified in symptomatic carriers. Purple bars indicate protease activities of missense HTRA1s identified only in CARASIL patients. I-bars indicate standard errors (SE). Statistical comparisons of protease activities between WT and each missense HTRA1 protein were performed with one-way analysis of variance followed by the Dunnett's *post hoc* test. ^***^P < 0.0001 for protease activities of each HTRA1 relative to WT. ^##^*P* < 0.0001 for protease activities of HTRA1 relative to WT.

**Figure 3:**
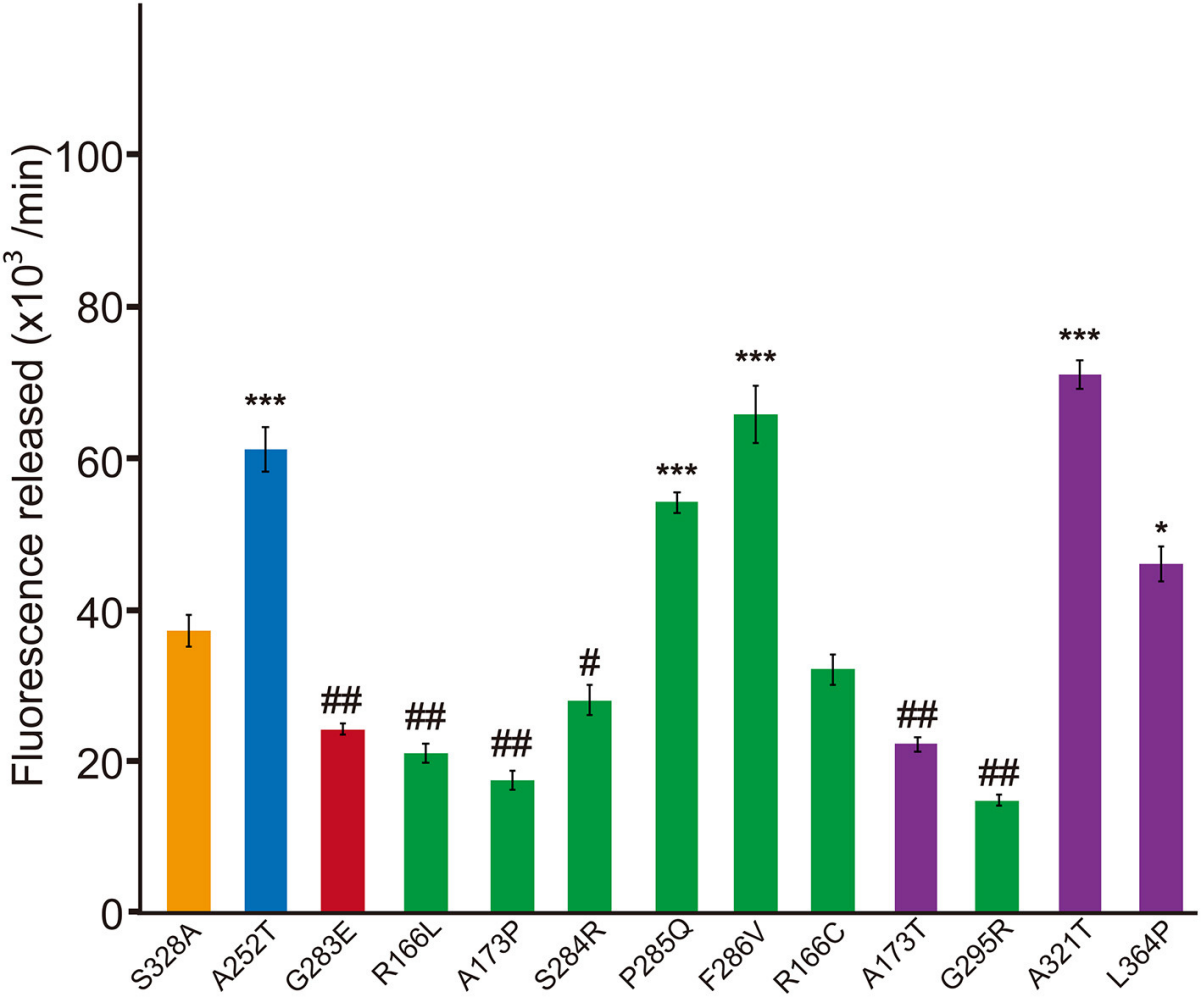
Dominant-negative effects of missense HTRA1s identified in symptomatic carriers. Protease activities of mixtures of each missense HTRA1 with WT calculated from the slope of the linear portion of normalized fluorescence vs. time (30, 60, and 90 min) plots. Orange, S328A/WT, a positive control for a dominant-negative effect. Blue and red bars indicate protease activities of A252T/WT and G283E/WT, respectively, negative and positive controls for dominant-negative effect, respectively. Green bars, missense HTRA1s identified in symptomatic carriers. Purple bars, missense HTRA1s found only in CARASIL patients. I-bars indicate standard errors (SE). Statistical comparisons of protease activities between each mutant HTRA1/WT and S328A/WT were performed with one-way analysis of variance followed by Dunnett's *post hoc* test. ^***^P < 0.0001; ^*^P < 0.05 for increases in protease activities for each HTRA1/WT relative to S328A/WT. ^##^*P* < 0.0001; ^#^*p* < 0.05 for differences for HTRA1/WT mixtures relative to S328A/WT.

The authors apologize for this error and state that this does not change the scientific conclusions of the article in any way. The original article has been updated.

## Publisher's Note

All claims expressed in this article are solely those of the authors and do not necessarily represent those of their affiliated organizations, or those of the publisher, the editors and the reviewers. Any product that may be evaluated in this article, or claim that may be made by its manufacturer, is not guaranteed or endorsed by the publisher.

