# Supplementary figures and images for: HTRA1 Mutations Identified in Symptomatic Carriers Have the Property of Interfering the Trimer-Dependent Activation Cascade
Source: Front Neurol. 2019 Jun 28;10:693. doi: 10.3389/fneur.2019.00693 (PMC6611441; doi:10.3389/fneur.2019.00693)

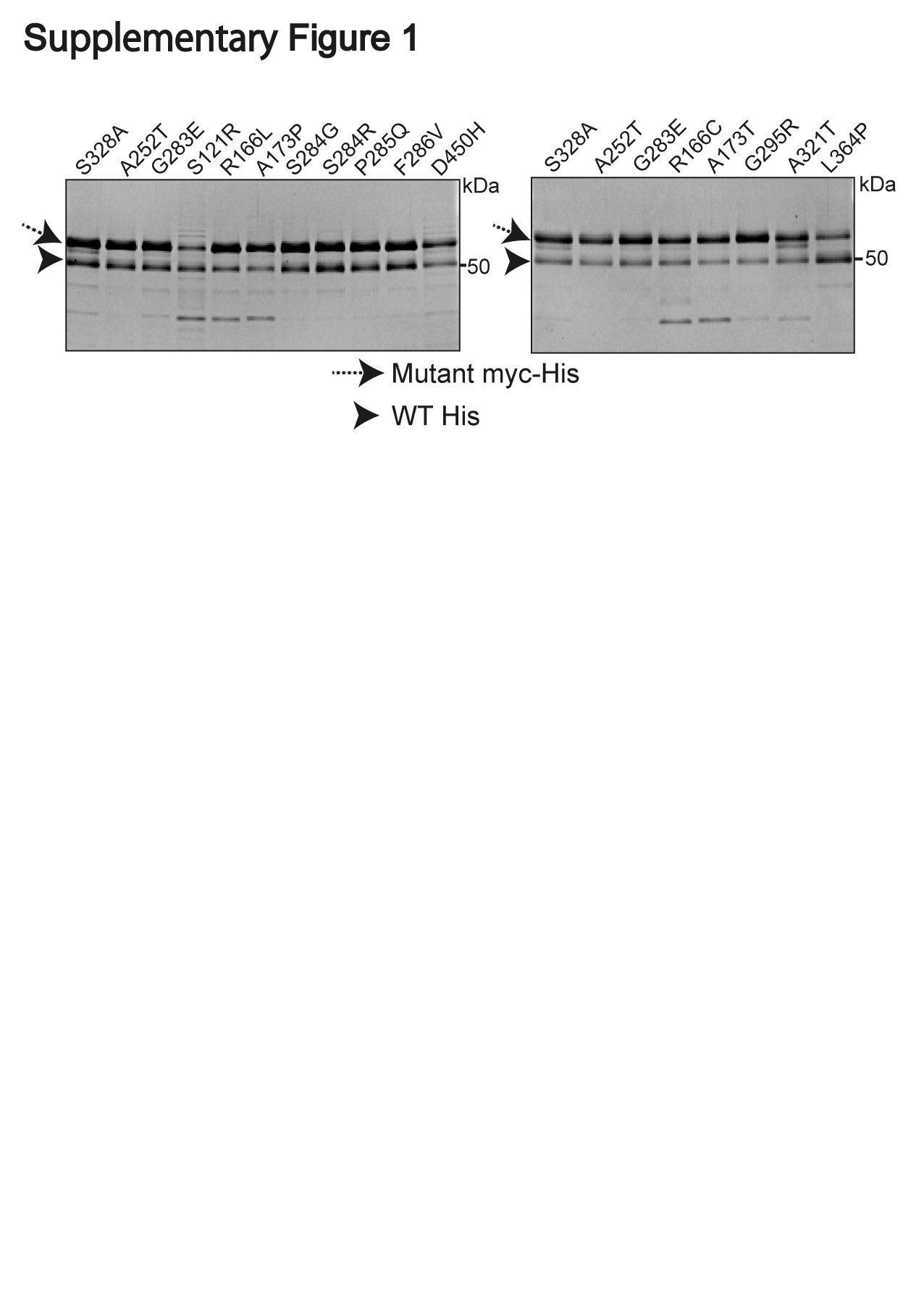

Supplement: Supplemental Figure 1 — Mixtures of missense HTRA1 proteins with WT protein. Mixture of each missense HTRA1 and WT protein evaluated by SDS-PAGE stained with SYPRO® Ruby. Broken arrows indicate the full-length band of missense HTRA1s tagged with myc-His6. Arrowheads indicate the full-length band of WT HTRA1 tagged with His6. [file Image_1.tif]
