# Supplementary material for: HTRA1 Mutations Identified in Symptomatic Carriers Have the Property of Interfering the Trimer-Dependent Activation Cascade
Source: Front Neurol. 2019 Jun 28;10:693. doi: 10.3389/fneur.2019.00693 (PMC6611441; doi:10.3389/fneur.2019.00693)
Supplement: Supplemental Table 1 — Summary of the clinical features of symptomatic heterozygotes with HTRA1 mutations. [file Table_1.docx]

**Supplemental Table 1. Summary of the clinical features of symptomatic heterozygotes with HTRA1 mutations.**

| Mutation No | 1 | 2 | 3 | 4 | 5 | 6 | 7 | 7 | 7 | 8 | 9 | 10 | 11 | 12 | 12 | 12 | 12 |
| --- | --- | --- | --- | --- | --- | --- | --- | --- | --- | --- | --- | --- | --- | --- | --- | --- | --- |
| Amino acid change | S121R | A123S | R133G | S284G | D450H | R166C | R166L | R166L | R166L | A173P | S284R | P285Q | F286V | G295R | G295R | G295R | G295R |
| Decreased protease activity | − | − | − | − | − | + | + | + | + | + | + | + | + | + | + | + | + |
| Sex | M | M | F | F | M | M | M | M | F | F | F | M | M | M | M | M | M |
| Age at onset (yrs) | 56 | UK | UK | 62 | 68 | 31 | 66 | 55 | 60 | 65 | UK | 50 | 49 | 65 | 67 | UK | UK |
| Age at diagnosis (yrs) | 66 | 50 | 58 | 62 | 72 | 31 | 69 | 64 | 65 | 72 | 49 | 55 | 55 | 73 | 71 | 63 | 49 |
| Age at MRI (yrs) | 62 | 50 | 58 | 62 | 70 | 31 | 69 | 64 | 60 | 72 | 49 | 50 | 55 | 73 | UK | UK | UK |
| HT | − | + | − | − | + | − | − | − | + | + | + | − | − | − | + | − | − |
| Initial neurological symptoms | balance impairment | seizures | headaches | stroke | stroke | vertigo | cognitive impairment | depressive mood | cognitive impairment | balance impairment | headaches | stroke | stroke | cognitive impairment | cognitive impairment | none | none |
| Stroke | − | − | − | + | + | − | + | − | + | − | − | + | + | − | + | − | − |
| Cognitive impairment | + | − | − | − | + | + | + | + | + | + | − | + | + | + | + | − | − |
| Gait disturbance | + | − | − | − | + | − | + | − | − | + | − | + | + | + | − | − | − |
| Alopecia | − | − | − | − | − | + | − | − | − | − | − | − | − | − | − | − | − |
| Spondylosis deformans | − | − | − | − | − | − | − | − | − | − | − | − | − | − | − | − | − |
| Severe WMLs | - | - | - | + | + | + | + | + | + | + | + | - | + | + | + | - | - |
| Country | France | France | France | France | France | Greece | France | France | France | France | France | France | France | Italy | Italy | Italy | Italy |

UK indicates unknown. + indicates present; and −, absent. WMLs indicate white matter lesions. Severe WMLs was defined as confluent WMLs.

References for the amino acids are:　S121R, A123S, R133G, R166L, A173P, S284G, S284R, D450H, P285Q, F286V — Verdura et al., 2015; R166C — Bougea et al., 2017;　G295R — Di Donato et al., 2017
